# Recent Trends, Technical Concepts and Components of Computer-Assisted Orthopedic Surgery Systems: A Comprehensive Review

**DOI:** 10.3390/s19235199

**Published:** 2019-11-27

**Authors:** Jan Kubicek, Filip Tomanec, Martin Cerny, Dominik Vilimek, Martina Kalova, David Oczka

**Affiliations:** Department of Cybernetics and Biomedical Engineering, VSB-Technical University of Ostrava, FEECS, 708 00 Ostrava-Poruba, Czech Republic; filip.tomanec@vsb.cz (F.T.); martin.cerny@vsb.cz (M.C.); vilimek.dominik@gmail.com (D.V.); martina.kalova@vsb.cz (M.K.); david.oczka@vsb.cz (D.O.)

**Keywords:** CAOS, fluoroscopic navigation, image-based navigation, imageless navigation, medical image processing

## Abstract

Computer-assisted orthopedic surgery (CAOS) systems have become one of the most important and challenging types of system in clinical orthopedics, as they enable precise treatment of musculoskeletal diseases, employing modern clinical navigation systems and surgical tools. This paper brings a comprehensive review of recent trends and possibilities of CAOS systems. There are three types of the surgical planning systems, including: systems based on the volumetric images (computer tomography (CT), magnetic resonance imaging (MRI) or ultrasound images), further systems utilize either 2D or 3D fluoroscopic images, and the last one utilizes the kinetic information about the joints and morphological information about the target bones. This complex review is focused on three fundamental aspects of CAOS systems: their essential components, types of CAOS systems, and mechanical tools used in CAOS systems. In this review, we also outline the possibilities for using ultrasound computer-assisted orthopedic surgery (UCAOS) systems as an alternative to conventionally used CAOS systems.

## 1. Introduction

The musculoskeletal system plays an essential role in human movement. This complex system includes the bones of the skeleton, cartilages, ligaments and the binding tissues which connect the organs together. This system is primarily focused on forming and supporting the human body, maintaining stability and performing movement. Musculoskeletal orthopaedical disorders and diseases usually cause severe long-term disability. As a result of these facts, the functionality of the musculoskeletal system and the severity of its disorders are crucial factors in human health and mobility [1,2,3].

When treating and providing therapy for the musculoskeletal system, traumatological and orthopedic surgery is conventionally used. In such procedures, the surgical steps—including ligament reconstruction and cutting and drilling of the bones—must be performed as precisely as possible. This accuracy predetermines the postoperative results; however, it also optimizes the risk factors of postoperative complications [3]. Surgeons often must cope with decreased visibility of the surgical tools when performing surgery. This phenomenon makes the surgical procedure more complicated, and it can be one of the significant factors affecting surgical accuracy. Recent trends in orthopedic surgery aim at minimizing invasiveness. Computer-assisted orthopedic surgery (CAOS) systems, which are used for the navigation, give surgeons access to real-time feedback on the surgery in progress by displaying a virtual image of the site of surgery on a computer device. This real-time tracking can be likened to the Global Positioning System (GPS), which provides drivers with instructions while driving [1,3].

**Figure 1:**
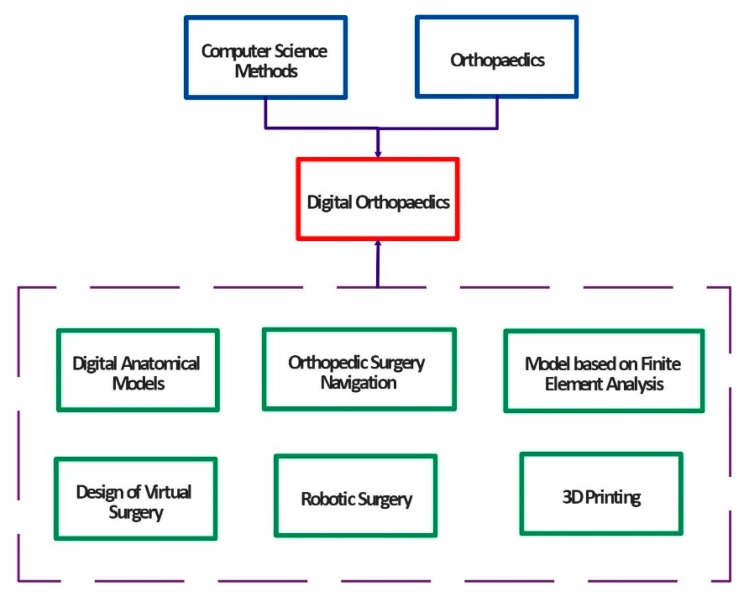
Basic structure and components of digital orthopaedics [4].

Within the development of the modern computing methods in medicine, digital techniques represent a significant role in improving clinical diagnosis, therapy, and consequent rehabilitation procedures. Application of the smart methods from computer science and digital techniques in the clinical orthopaedics facilitate and bring novel possibilities in medicine. A combination of the digital techniques and clinical procedures lead to a modern trend which is called the digital orthopaedics. Figure 1 shows the basic concept and components of digital orthopaedics [4,5].

**Figure 2:**
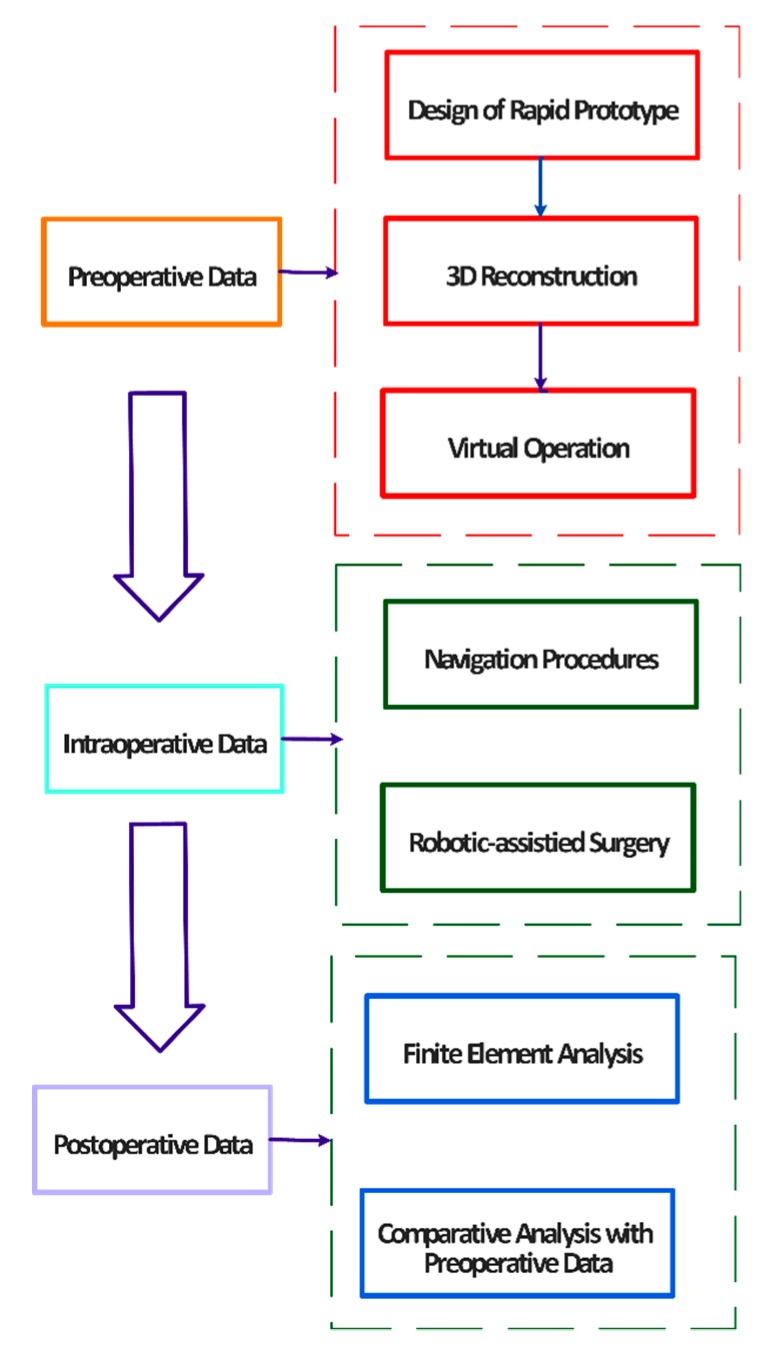
General application of digital [4].

At present, science and modern intelligent methods are growing and developing at a high speed. Digital orthopaedics represents a multidisciplinary area, involving the clinical medicine, and cooperation with the technical specialist from computer science, mechanical and material engineering and others related areas. The digital orthopaedics mainly helps to clinicians improve and manage the whole surgical procedures. These surgical procedures commonly include a preoperative analysis and decisions, intraoperative procedures and postoperative management. Individual technologies which are used in the digital orthopaedics involve the virtual surgical planning, computer-assisted navigation within an operation, virtual operation training and others. Basic research of the digital orthopaedics is based on the digital orthopaedics anatomy and biomechanical research. These two procedures provide an essential support for individual applications in the digital orthopaedics. Digital orthopaedics is also used for training and education procedures of trainee physicians in the form of virtual instruments with the goal to improve their skills. The whole application procedure describing individual components of the digital orthopaedics is demonstrated on the Figure 2 [4,5,6,7].

Computer-assisted orthopedic surgery systems represent a recent concept in the orthopedic surgery. From the perspective of orthopedic clinical practice, CAOS systems have frequently been used in arthroplasty and spinal surgery [1,2]. In arthroplasty, CAOS systems allow for modelling of the patient’s anatomical structures including acetabular abduction, flexion and hip offset [8,9]. By using CAOS systems in spinal surgery, it is possible to perform more accurate insertion of pedicle screws [10,11,12]. This fact is more important in the case of spinal deformity and scoliosis, where normal spinal anatomy is deformed and therefore it is more complicated to find an optimal location and pathway for the pedicle screw. Recently, there has been increased interest in using CAOS systems in cases of orthopedic trauma [13,14,15]; here CAOS systems are used in order to improve the accuracy of implant placement in cases of pelvis and acetabular fractures [16,17].

In this paper, we present a complex and comprehensive overview of the components and techniques of the CAOS system. We are aware that the CAOS represents a multidisciplinary area, involving the clinical procedures, computer science, mechanical and material engineering. In the comparison with other related review papers of CAOS systems which are mostly focused on types of the CAOS systems, we provide a broad spectrum of the methods which are essential for the CAOS systems. Section 2 is dedicated to the image processing techniques and procedures for manipulation with the clinical images used in CAOS systems. These techniques mainly include the image enhancement, image segmentation, image registration, and definition of anatomic landmarks. Consequently, we describe individual concepts of CAOS systems, including 3D image-based navigation, Fluoroscopic navigation, imageless navigation, and the ultrasound-based navigation. Lastly, we outline a role of the medical robots for the bone fracture reduction. Section 3 is dedicated to the biomechanical components of the CAOS systems. In this section, we summarize the utilization of pedicle, intraosseous, Schanz and cannulated screws, and complex shaped implants for the CAOS applications. The consequent part of this section is focused on the materials of the orthopedic implants.

## 2. Essential Components and Types of CAOS Systems

Each CAOS system comprises a complex navigation system, which is made up of several components. The core of this system is the computer navigation unit, incorporating special software tools [18,19]. This unit is connected with an optical tracking camera and LED trackers: an LED is attached to the image source, which is the C-arm, and another LED is attached to the surgical instrument and a monitor [20,21].

CAOS systems based on image navigation include sophisticated algorithms, reported in Section 2.1, Section 2.2, Section 2.3 and Section 2.4. These procedures utilize the essential elements of the software equipment of the CAOS systems. A process of the data acquisition serves for obtaining the clinical images, containing the anatomical information about the bones and other interested tissues for the image-based CAOS systems. The first part of the clinical image processing is the image enhancement. In Section 2.2, we report essential procedures, allowing for an enhancement of the image parameters to ensure deeper contrast and related spatial image parameters of the bones. This fact predetermines better accuracy of the bone’s identification and modeling, and thus effectivity of the whole image-based navigation. In this section, we also devote to the techniques which are applied in the CAOS systems for the noise suppression to achieve smooth areas of the bones. Section 2.3 is dedicated to the image segmentation methods, enabling a decomposition of the image area into individual objects. This procedure is essential for the bones modeling in the image-based CAOS systems. Segmentation methods perform extraction of the bone structures and consequently enable their manipulation and visualization in the form of the virtual model which is used for operative planning. We present conventionally used segmentation techniques for CT and MR images for the CAOS systems. Section 2.4 is dedicated for the methods of the image registration in the CAOS systems. The image registration provides a geometrical transformation between a therapeutic and virtual object to display the final effect of the localization, respecting the virtual representation. Section 2.5 is dedicated to the definition of the anatomic landmarks which are essential for building the registration systems. These parameters serve as the reference points. Landmarks are also important in the imageless navigation for definition of the anatomic frames, corresponding with the spatial features, like is the position and orientation.

### 2.1. Image Data Acquisition

For the image-based CAOS systems, the clinical images, carrying anatomical information are crucial for the surgical navigation. These images are used for the bones modeling and guiding surgeons within the operation. The first important element of a CAOS system is the acquisition of clinical images reporting the patient’s anatomy and pathology (in most cases, fractures). Such images serve as input data for the computer analysis, and they guide surgeons during the surgical procedure. In orthopedics and traumatology, data are acquired from several sources including 2D/3D fluoroscopy, CT scans, and in some cases also MR images [22,23,24,25,26,27].

### 2.2. Image Enhancement

The first step of clinical image processing in image-based CAOS systems is the image enhancement. Procedures of the image enhancement are generally aimed on improving of the image parameters with the goal of the noise suppression and optimization of the image spatial area. These procedures are essential for the consequent image segmentation, making virtual bone models for guiding the orthopedic surgery. A preciseness of these models is depended on the quality of the input image information. Image-guided CAOS systems rely on the quality of the images they provide [28,29]. For each clinical procedure, surgeons work with different image data, which are acquired from different imaging systems (CT, MRI, etc.) and have varying levels of spatial quality [30,31,32,33,34,35,36]. For this reason, image enhancement techniques are often employed to improve the quality of the clinical image. This procedure is very important for the subsequent modelling of the bones and other tissues, as it can be expected that poorer image quality will lead to incorrect recognition and identification of the bones [37,38,39]. Image enhancement techniques are mostly focused on contrast enhancement in order to highlight the boundaries between the object of interest and adjacent tissues [40,41]. The image enhancement techniques are also used for the image noise suppression. This task is conventionally done by using the smoothing filters, suppressing a high-frequency noise. For this task, the statistical filtering based on the Gaussian, average or median filter is routinely used. Image data used in the CAOS systems may be generally influenced by the additive noise, deteriorating the spatial image area. Therefore, the bone tissues which are essential for the CAOS systems are influenced by this image noise. Applying the smoothing filtering makes the spatial image information fluent and increase the preciseness of the modeling. It should be noted that the accuracy of the bone modeling in CAOS systems is directly linked with the spatial image quality [40,41].

Image interpolation plays an important role in image enhancement. This technique involves the use of image scaling to improve image resolution [42]. Types include adaptive sub-pixel interpolation, interpolation based on wavelet transformation (high-frequency sub-bands), linear/cubic interpolation, vector quantization, and dual tree-complex wavelet interpolation [25,26,27]. In most cases, the purpose of the interpolation procedure is to enlarge the image based on the scale factor. This factor is derived from statistical features of neighboring pixels, such as the median or mean. Generally, image interpolation transforms the original pixels from low-resolution images to high-resolution frames with sub-pixel shift information. Interpolation is primarily important when performing image zooming. When zooming, we extract a part of the image that has fewer pixels when compared with the original image area. Interpolation estimates the surrounding pixels and thus enhances the image resolution [43,44].

A significant issue in image enhancement is contrast analysis. In the image-based CAOS system is the contrast information frequently used for recognition between the bones and the image background for making of the virtual bone models. Contrast results from the difference between the intensities of two adjacent pixels in a clinical image. A low-contrast image is a consequence of non-uniform lighting conditions, non-linearity of intensities, or the insufficient dynamic range of the imaging sensor. Contrast stretching (CS) is a very popular method of contrast enhancement. This method enhances contrast by stretching the range of intensity values. CS performs contrast equalization in the image area by using simultaneous adjustment of each monochromatic value in the darkest and lightest portions. When comparing CS with other contrast enhancement algorithms, conventional techniques are applied via a linear scaling function for all the pixels, which results in more sensitive results [45,46,47].

Histogram equalization (HE) is a procedure that transforms a clinical image with non-uniform intensity levels to create a resulting image with uniform intensity levels. The main task of HE is to align the image intensities as much as possible. HE is a very popular method as it offers numerous benefits, such as greater computational simplicity and comparatively better performance; for this reason, HE is often employed in computer vision algorithms. In the image-based CAOS systems, the HE is used for the optimization of the intensity distribution with the goal to differentiate bones from the image background, when the spatial area is impaired by worse intensity distribution [48,49].

Besides their spatial area, clinical images are also characterized by their frequency domain. The spatial frequencies are conventionally used for the bones localization. Therefore, emphasizing the spatial frequencies is crucial for an optimization of the bone intensities distribution. Alpha rooting (AR) combines both spatial and frequency information enhancement with the goal of maximizing the advantages and at the same time minimizing the limitations of both types of information. AR is typically used to emphasize high-frequency image content by using the power law and log transform. This technique transforms a narrow range of input intensities to a wider output range, enabling AR to enhance contrast and retain clinical information and details. The main benefit of AR is that it maintains clear edges (image details) and distinguishable features [49,50,51].

Another popular technique for image enhancement is the use of wavelet transformation (WT). This method is popular due to the wide range of wavelets possessing specific features allowing for image decomposition and thus the extraction of important image features. For the 2D implementation, separable wavelets are used. These 2D wavelets are capable of approximating vertical and horizontal edges. WT is frequently used for tasks such as image denoising, contrast enhancement and feature extraction [52].

### 2.3. Image Segmentation

A key procedure in the identification of the clinical objects is image segmentation. Segmentation procedures are used in the image-based CAOS systems for making the virtual models of the bones. The bone models, in the comparison with native images, bring opportunity to interactive manipulate with the bones, extract the geometrical parameters and simulate bone interaction with the mechanical tools within the orthopedics surgery. In computer vision, image segmentation is a process that partitions the clinical image into finite image segments. We can distinguish binary segmentation (with two recognizable regions) and multiregional segmentation (which is represented by labels of more than two tissues) [53,54]. It is generally assumed that the segmented object is represented by a finite set of labelled pixels. In 3D bone imaging and analysis, computer tomography (CT) plays the most important role. This is because ordinary image thresholding is sufficient to detect and identify the bone tissue. This method is based on the higher attenuation of X-rays by bone tissues compared with soft tissues. 3D segmentation models enable spatial observation of fractured bones and the registration of other images by using bones and the skeleton as a region of interest [54,55]. Nevertheless, only cortical bone has the high density which is required to differentiate it from adjacent soft tissues. Unfortunately, many bones are porous (spongy); such bones provide structural support and flexibility. These spongy bones are characterized by low X-ray attenuation, and their density differs only slightly from the density of soft tissues. This phenomenon is particularly observable in elderly patients, i.e., a population that frequently suffers from bone weakness. In order to perform proper bone identification, it is necessary to achieve a substantial contrast between bones and soft tissues. Manual image segmentation is a long-established method used in order to extract the geometrical features of bones [56,57]. Unfortunately, this process is a lengthy one, and is associated with high variability. Modern trends in image segmentation are focused on automatic image segmentation. Such a process ideally involves the autonomous identification of the analyzed bone from the soft tissues by means of the binary segmentation model [57].

Automatic, semi-automatic and interactive segmentation methods are conventionally used in CAOS systems. These methods utilize imprecise user input, yet they provide much more precise delineation of bone shape. In the following text, we describe several segmentation techniques which are suitable for use with bone and bone cavity segmentation in CT images [58,59].

Image thresholding is a basic technique for separating the object of interest from the rest of the monochromatic image (the image background). This operation is performed by labelling all the voxels lying below and above a certain intensity threshold. In CT images, the HU scale is used as a guideline for selecting the threshold separating individual tissues. We can distinguish several types of thresholding, including global and hysteresis thresholding [60,61,62].

Global thresholding is the simplest thresholding method, and it is widely used for clinical image segmentation. This method is capable of extracting bright objects (bones) from a darker background (soft tissues). The main limitation of this method is its inability to separate objects with overlapping intensities. This complication is manifested mainly in the case of spongy bones and in the presence of noise in the image [29,63].

Hysteresis thresholding is a more sophisticated thresholding method which takes into account intensity values and spatial relationships between individual voxels. This method is much more effective at separating lower-intensity bones from image noise and soft tissues. Hysteresis thresholding uses the dual threshold to extract high-intensity bone voxels and subsequently to perform a connectivity analysis in order to find all the lower-intensity bone voxels which are connected to high-intensity bone voxels [64,65,66].

Another type of segmentation involves placing user-defined markers (seeds) as input guiding points for tissue segmentation. A typical seed-based approach is random walk segmentation. This algorithm calculates the probability for each voxel that a “random walker” moving from a voxel will reach a seed label. In this method, the clinical CT image is represented by a weighted graph: G=(V,E), where the vertices *V* represent the voxels and the edges *E* stand for the connections between adjacent voxels in a 6-connected neighborhood. Each edge between two neighbor vertices is described by a weight, which is described by its magnitude. This method creates a sparse linear system composed from the graph and seed nodes. Individual labelling is performed for each voxel with the highest probability. An advantage of this method is its capacity for iterative editing by adding further seeds and running the solver again [67,68].

Mesh segmentation algorithms are used to segregate polygon meshes into individual sub-components. These algorithms are applied in 3D CT modelling and CAD applications. By using mesh segmentation, we obtain an intuitive sketch or an interface with landmarks. This operation enables objects of interest to be easily marked, so it is possible to interactively edit the segmentation results [69,70].

A very popular alternative segmentation method involves deformable surface models. This approach to segmentation utilizes the elastic curves that operate in the spatial image domain. Such a curve undergoes an evolution when its geometrical features are deformed to the bone edges. This evolution is driven by a minimization of the cost function. The main benefit of this approach is its ability to segment different geometrical shapes, which display a high level of robustness to image noise and artefacts. As a consequence of these features, surface models are an effective tool for bone segmentation from CT images [71,72].

The final important method is segmentation evaluation, which reveals the accuracy of segmentation. This process is very important within the design of the software part of the image-based CAOS system. Based on the objective evaluation, we have information about the segmentation accuracy and robustness for particular clinical using. This fact declares effectivity of using segmentation method for making the virtual bone model. Segmentation evaluation is a process which shows the performance of individual segmentation methods for a specific application. It is important to be aware that segmentation accuracy is linked with specific applications; for instance, we cannot expect that the same bone will be displayed with the same image features when different imaging methods are used. When conducting segmentation evaluation, we asses three parameters: precision, accuracy and efficiency [71,72,73].

The precision of the segmentation process expresses its degree of repeatability. This metric evaluates how sensitive a particular segmentation method is. Segmentation accuracy expresses the closeness of the segmentation to the ground truth image. Since there is no genuine ground truth segmentation for real CT data, manual segmentation is usually performed by clinical experts. In order to avoid subjectivity, multiple manual segmentation of the same object is standardly performed by various clinical experts [73]. Segmentation accuracy is normally evaluated by the Jaccard index or the Dice coefficient. Volume similarity is often evaluated by Bland-Altman plots. The third and final parameter is segmentation efficiency, which expresses the practical utility of the method with regard to time and cost. This parameter is evaluated by recording training time, user interaction time and computation demands. Based on these values, a function evaluating cost based on time factors is defined [73,74].

### 2.4. Image Registration

Medical image registration (MIR) is one of the crucial steps in the image-based CAOS system. MIR is conventionally used for aligning preoperative and postoperative images, or for alignment of therapeutic and virtual objects. During this procedure, two or more individual images are matched and aligned into one single output image which provides more valuable information [75,76]. MIR is based on the estimation of an optimal transformation which is assumed to best match the object of interest. Generally, in MIR one of the source images is modified and adjusted by using geometric transformations, such as translation, rotation, scaling or deformation relative to a fixed image (the target image). Image registration is an important procedure for processing images from the same imaging system in different time-dependent frames due to the movement of the object (caused by the patient’s movement or changes in the patient’s position). The registration of the images acquired from different image sources is of substantial importance because it provides different representations of the same structure in different images [77]. Currently, one of the challenges faced by MIR systems is the problematic availability of precise, computationally efficient, and yet also clinically acceptable registration techniques. Although MIR is capable of providing useful clinical information from different clinical images, we have to strike a balance between accuracy and efficiency. Besides these issues with MIR, there are also other challenges which should be taken into account—including the possibility of automatic image registration, the detection of bone landmarks, the registration of multimodal images, and robustness to outliers in clinical images. The following text presents a brief categorization of registration methods [78,79,80].

Registration is based on the transformation of computed reference points from the source image (moving image). These points are subsequently mapped and matched with the target image by using various transformation methods, including rigid and non-rigid transformation. Rigid transformation is based on the translation and rotation of a moving object (the source image) with respect to the coordinates of the target image. A specific feature of rigid transformation is the fact that it retains the same geometric features, i.e., the same angles and distances between individual points. Affine transformation performs scaling, shearing and rotation. This method is accurate and robust against outliers and image artefacts. Another benefit of this method is that it enables the easy alignment of clinical images obtained from multiple sources [81,82].

Domain transformation performs the registration procedure by means of a transformation in the image domain. A benefit of domain transformation is that it can be applied either to the entire image area or to sub-parts of the image. In this type of registration, pixel information is transformed into another domain with the goal of obtaining more accurate information. The domains of registration are commonly divided into global and local registration. Local transformation concerns local geometric features of the source and target images. Global transformation concerns all the image parameters from the entire image area. This type of registration is affected by any modification of parameters. Global transformation may be effectively conducted by using local parameters affected by image noise and artefacts, because it suppresses these portions of the signal as part of the image preprocessing methods which are used in the domain of registration transformation [82,83].

A very important factor in image registration is image dimensionality, as transformation is carried out either by using the image coordinate system or the image and the physical phase. Based on the dimensionality, the image registration can be spatial, or time series can be registered. Depending on the number of geometrical dimensions, we distinguish 2D/2D, 2D/3D and 3D/3D registrations. Analysis of dynamic sequences of clinical images based on time series registration enables bone features to be tracked over time [83,84].

Another popular type of transformation is registration based on an optimization procedure. In this method, the registration process is performed repeatedly in order to optimize the transformation parameters. Here the registration is generally based on maximizing the similarity between the analyzed images. An optimization procedure iteratively recalculates and adjusts registration parameter differences between source and target images. The registration performance is determined by the accuracy of the optimization procedure. A crucial task of the optimization procedure is to select appropriate parameters which possess a high level of similarity. Finally, the obtained parameters are used to achieve the best matched image based on similarity metrics [83,85,86].

Generally, image registration may be performed in three ways: semi-automatic techniques, fully automatic techniques, and interactive registration. Interactive segmentation is used in clinical applications when the preoperative image space needs to be correlated with real-time physical space [85,86]. Interactive registration involves the following procedure. In the first step, the clinical images are obtained by a scanner (MRI or CT); the region of interest (RoI) is thus interactively selected based on the expert’s experience. In the next step, the interoperable images are matched and registered with the preoperative images [83]. The main benefit of this type of registration is the dynamic matching and tracking of the RoI. Automatic registration of multimodal and structural images by automatic and semi-automatic selection of the RoI remains a challenging research issue. These procedures are very important as they enable the detection (without user interaction) of similar regions from preoperative and intraoperative images [86,87,88,89,90].

A very popular method for aligning bone models acquired from CT data is iterative closest point (ICP) registration. [91] In this approach, we utilize an initial coarse alignment of the models [92]. The registration process is iteratively adjusted via the following sequence of steps:

- Definition of the closest corresponding point on the target model for all the points and vertexes of the source models.
- Definition and computing of the rigid-body transformation based on geometric transformation, including translation and rotation. The purpose of this procedure is to minimize the average distance between corresponding points.
- Application of the transformation for all the source points.
- Repetition of the previous steps until the average distance is lower than the preset threshold.

ICP registration needs an initial reasonable estimation to reach convergence. This is one of the essential limitations of the ICP method. Another issue with the method is its sensitivity regarding image noise; the algorithm is not sufficiently robust against noise [93,94,95].

### 2.5. Definition of Anatomical Landmarks

**Figure 3:**
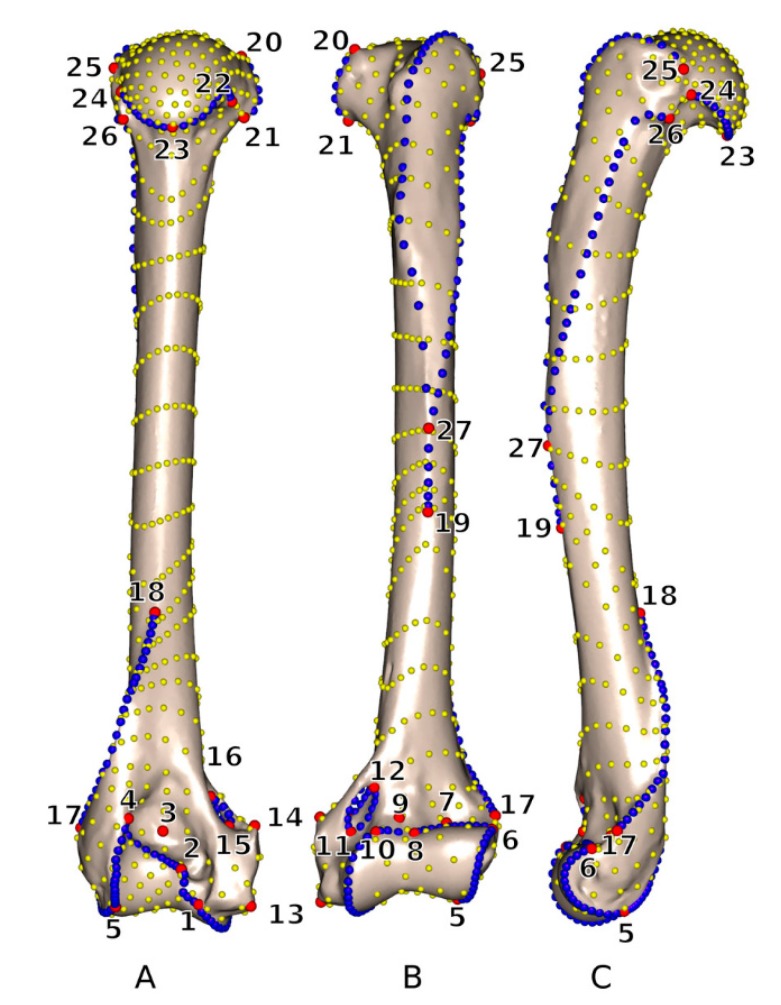
3D view of humerus which shows the location of the 27 anatomic landmarks and 18 curves which are used to quantify the humeral shape: (A) caudal, (B) cranial and (C) lateral view. Red marks indicate the anatomical landmarks, blue marks represent curve sliding semi-landmarks and yellow is surface sliding semi-landmarks. [102].

To build registration systems, which are crucial for CAOS, it is essential to identify and detect reference parameters and landmarks (Figure 3). Landmarks allow for the definition of morphological parameters, including distances, curvatures and angles. Such parameters are important for recognizing and distinguishing dysplastic from normal morphological structures [95,96]. Surgical navigation systems rely on landmarks, which serve as reference points. Image-based navigation systems require image registration, so landmarks are very important. Imageless navigation systems also utilize landmarks in order to define anatomical frames, which are related to spatial features including the position and orientation of the reference frame being fixed to the bones [96,97]. Finally, there are various landmarks which define the intersection in ligament reconstruction. Anatomical landmarks are defined in various ways. In the case of live subjects, they are commonly defined by manual palpation and transformed into a digital format using a probe. Additionally, in surgical systems, landmarks are often defined based on the kinematic analysis of the joint. This method of analysis focuses on the centre of the hip, knee and ankle [97].

Some imaging methods make use of virtual landmark identification. In this context, 2D X-ray images are the most conventionally used images in orthopedics. However, X-ray images suffer from overlaps of anatomical structures and undesirable distortions. Therefore, CT and MR techniques are longer-established methods of obtaining detailed images [97,98]. One substantial benefit of these image modalities is that they enable 3D reconstruction from individual slices. In each case, we have to consider intra- and inter-observer variability when modelling landmark-based anatomical parameters. Variability is primarily caused by manual marking of the landmarks. This approach is subjective, relying on the knowledge and experience of the surgeon performing the task [97].

Due to the above-mentioned variability, automatic systems for landmark definition are becoming increasingly popular. We can verify the accuracy and robustness of these systems based on the gold standard defined by clinical experts. For automatic systems, heuristic methods can be used. Such methods are able to extract lateral and medial epycondyles, the transepicondylar axis, inferior and posterior locations of condyles, the femoral condylar axis, and morphological features of the femoral head (including the centre, radius and other features) [98,99,100]. Heuristic algorithms have been tested and evaluated on 100 CT scans of femurs with normal and pathological features. Generally, these automatic methods offer several benefits when compared with manual methods. Firstly, they reduce computing times and allow for immediate digital image processing. Secondly, they eliminate inaccuracies caused by the subjective factor (observer variability) [100,101]. Lastly, we can evaluate the accuracy and robustness of automatic methods against the gold standard. Nevertheless, we need to consider the use of the automatic method due to algorithm training. Such procedures need to be trained on extensive retrospective data; proper training is a prerequisite of valid results [102,103,104].

### 2.6. Introduction to Navigation Systems

Surgical navigation represents a visualization system providing clinically important information about surgical instruments and implants and their orientation to the target organ. Such systems conventionally utilize 3D position sensors for spatial orientation. These sensors are able to track the target organs, surgical tools and implants. A crucial step for any navigation system is a surgical plan providing positional information and incorporating information about the surgical tools. In clinical practice we distinguish several types of surgical planning. One type (generally termed volumetric image-based navigation methods) is based on the use of volumetric images from imaging systems such as CT, MRI or ultrasound imaging [105,106]. Another popular type (fluoroscopic-based navigation) uses intraoperative fluoroscopic images. The last type (imageless navigation) provides kinetic information on joint movement. Computer-assisted surgery (CAS) systems and computer-assisted orthopedic surgery (CAOS) systems, optical sensors and magnetic sensors are frequently used [106,107].

The positional information is acquired by using CCD cameras. These devices are based on infrared light emanating from a dynamic reference frame (DRF); such systems utilize infrared light-emitting diodes (LEDs) or light-reflecting markers. In surgical planning, the DRF is attached to the target bone and to the surgical tool, which can be tracked. Measurements obtained in this manner are rapid, and they are considered highly precise. The main advantage of such systems is that they enable the simultaneous tracking of multiple LEDs. Another technology involves the use of active markers, which require a power supply; electrical cords can present a disadvantage as they can obscure the surgical field. However, there also exist active wireless LED markers, which are battery-powered. Another issue is the magnetic sensors, primarily with regard to their accuracy. This accuracy can be limited by the presence of motors in the operating room or metallic instruments. In some applications, magnetic sensors are used for catheter tip tracking, though many orthopedic surgery systems use optical sensors [108,109,110].

A special part of the navigation system is implant and modeling of its interaction with the human body. For the fracture fixation, the various mechanical components are used, like is wire, stems, rods, screws and others. A very important issue of the implants is the biocompatibility. Recent trends lead to using biomaterials. A classification of the biomaterials is based on the structural criteria: metal, ceramic, polymeric, natural origin and composite. Concerning the prosthetics, it is used phenomenological analysis, and also the analysis of the internal prostheses, such is artificial joints. The important fact is that the interaction between implant and surrounding tissues must not cause the corrosion and degradation, secondary reactions in the human body and the implant instability. In manufacturing technologies of the implant materials we recognize the casting technology, stamping technology, material removal technology, and rapid prototyping technology [111,112,113].

### 2.7. 3D image-Based Navigation

**Figure 4:**
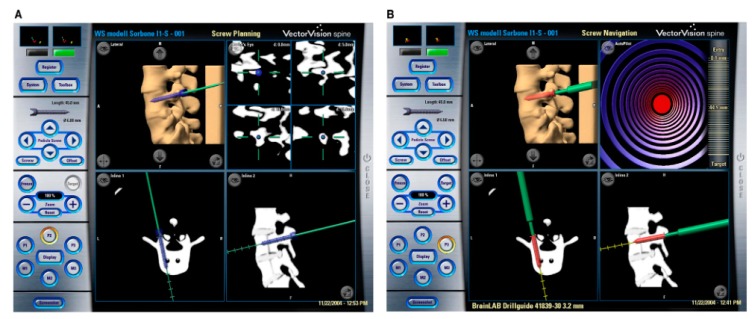
CT-based navigation feedback within the pre-operative planning (**a**) and (**b**) reports intra-operative navigation of the pedicle screw placement [3].

3D image-based navigation conventionally utilizes volumetric images. Clinical data are conventionally provided by CT, MRI and ultrasound images. Comparing the benefits of individual image modalities, CT is the most frequently used as it offers many advantages. First, CT imaging allows for high-resolution images with high contrast between bone and the surrounding soft tissues, a long scanning range and simultaneously a short scanning time [114,115]. Since the bones are displayed with substantial contrast, it is easy to perform an effective segmentation of the bones. This segmentation procedure generates a model of the bones, in which other structures are suppressed. Furthermore, by using high-speed CT scanners, it is possible to avoid the motion artefact of 3D skeletal models. Navigation systems of this type are successfully used for spinal pedicle screw insertion (Figure 4), hip arthroplasty (THA), pelvic osteotomy, total knee arthroplasty (TKA) and knee ligament reconstruction [115,116]. Particular imaging methods are selected depending on the aims of the navigation. Spinal pedicle screw insertion requires CT images with a slice pitch of 1 mm and a resolution of at least 0.3 mm. For joint reconstruction surgery, CT images with a slice pitch in the range 1–10 mm and image resolution of at least 0.6 mm should be used. When considering patient motion, we should be aware of motion artefacts. There are several models for detecting the distortion caused by patient motion in 3D skeletal models. Rod-shaped objects can be scanned along the body [117,118]. Alternatively, the scanned objects can be viewed from several directions. Such an approach allows for the assessment of the distortion. In 3D preoperative planning, the multi-slice method is used; the patient’s body is shown in three orthogonal planes (coronal, sagittal and axial). Subsequently, image overlaying is used. This procedure uses computer-aided design (CAD) files of the implants in conjunction with the CT images; this enables the orientation of the implants to be shown. By using this procedure, it is easy to create a surgical plan for the optimal placement of straight-shaped implants—such as the femoral components used in THA. The next method involves volume rendering and surface models; these methods also allow bone imaging in a 3D space. In total joint arthroplasty, 3D CAD files and surface models serve for the simulation of motion. However, one disadvantage of this method is the difficulty of adjusting the fitting of implants [119,120,121,122].

In total joint arthroplasty or the fusion of multiple spinal segments, we need to track and measure the alignment between bones, and also between the bone and the implant. For this task, standard coordinates of the target bones are used, which are calculated based on the anatomical landmarks. It is assumed that the zero positions of joints will not differ significantly among individual patients [123,124]. However, the zero positions of the hip and spine may be modified by individual body shape, ageing and spinal deformity caused by osteoporosis. Since the target implants and operating tools can be tracked by using DRFs attached to bones, these tools can be shown in the 3D volumetric images of the bones. This simultaneous visualization is performed by registration of the bones and tools and calibration of the implants. Registration involves matching the CAD files of the implants and surgical tools with their position relative to the DRFs [125]. Conventionally, we recognize two registration methods. In paired matching registration, it is assumed that the surgeon sets at least three landmarks in the preoperative images, and subsequently sets the corresponding three landmarks in the patient’s body during the surgery. The major disadvantage of this method is its non-reproducibility. In order to compensate for this non-reproducibility, reference markers (fiducials) are placed on the target bones before performing the volumetric imaging. This method is known as reference registration. The references are used to create a 3D reference model of the patient’s bone. The reference locations from the preoperative surgical plan are subsequently used for the bone position in the preoperative plan [117,118]. Regarding its surgical effectiveness, this method is accurate; however, it brings a further complication in the form of an additional operation (the placement of the reference markers). Another registration method involves shape-based surface registration. This method utilizes corresponding points; a corresponding point is defined as a computer model point placed at the nearest distance to a measured surface point. The algorithm is iteratively repeated in order to minimize the average distance between each measured point and its corresponding surface point. In mathematical terms, this algorithm uses the iterative closest point and the least square method [126,127]. An important part of the algorithm is the starting point for the registration. This task is performed by means of baseline registration using the paired point method. In the subsequent step, the registration algorithm uses several surface points for the final matching. Intraoperative surface data are acquired by using ultrasound probes with DRFs. The accuracy of this method depends on the accuracy of the computer model, and also on the quality of the intraoperative data.

When creating 3D volumetric models, other tissues (such as soft tissues) are suppressed in the segmentation model [128,129]. For instance, an accurate surface registration of the pelvis is performed by using a computer model generated with the threshold from 140 to 260 Hounsfield units. This thresholding should exploit the balance between the soft tissue noise and the bone surface effect. It is recommended to use at least 20 surface points to achieve an accurate surface registration of the spine or knee. Another important aspect is the dispersion of the individual points. These points should be obtained with dispersion in order to create YZ, XY and ZX planes of the target bone model. A further aspect is the presence of residual registration points. These points represent the difference between the intraoperative data and the preoperative models. A correlation can be found between the accuracy of the navigation and these residual points, but they are not the same [130,131].

From the clinical perspective, residues larger than 1 mm are not accepted—and this includes cases in which the target area is close to the data sampling area. The next step is the post-registration phase. In this phase, surgeons should verify the rigidity loss between DRFs and the target bones. This navigation is beneficial for the socket orientation in THA when compared with conventional mechanical guides. This type of navigation is also suitable for guiding the femoral components and selecting optimal modular parts for THA. Generally, the main benefit of in 3D volumetric navigation is its accuracy. Furthermore, intraoperative control by the surgeon is not necessary. However, 3D volumetric systems require longer computing time when compared with other systems, leading to longer preoperative preparation times [121,129,132].

### 2.8. Fluoroscopic Navigation

**Figure 5:**
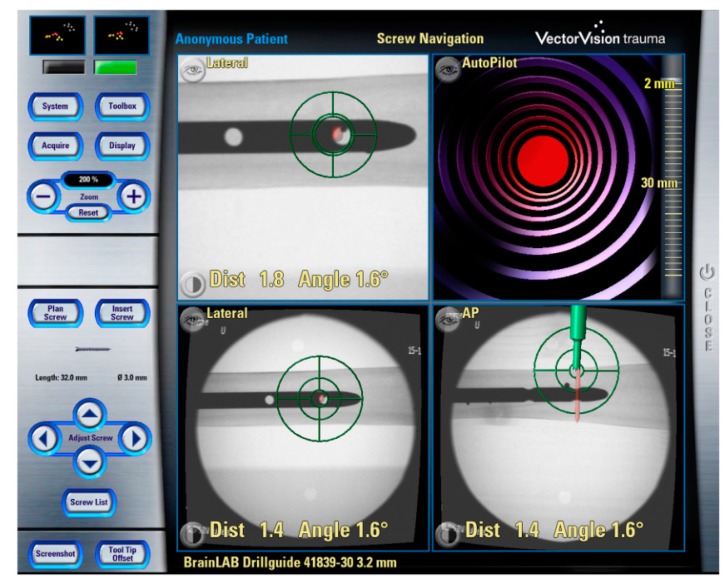
Fluoroscopy based CAOS system for distal locking of the intramedullary nail [3].

The main advantage of fluoroscopic navigation (Figure 5) is its lower cost compared with volumetric navigation. Based on fluoroscopic images, it is possible to create a guiding map which is used during the surgery [133,134].

The fluoroscopic images are generated by attaching DRFs to the C-arm and the target bones. This procedure determines the position of the image with respect to the patient. It utilizes CAD files containing implants and surgical tools with DRFs which are superimposed on the images without the need for a registration procedure [135,136]. A significant benefit of this type of system is that does not require registration; the computing time is thus reduced and the radiation dose is lower because the first several images are used repeatedly to guide the operative procedures. This system has conventionally been used for fracture reduction and fixation. In the case of the spine and pelvis, fluoroscopic 3D image-based navigation from multiple C-arm images is used [137]. However, comparing 3D C-arm images with CT images, there is a substantial difference in quality; the CT image quality is higher. To prevent image distortion, calibration markers are used, which are imaged simultaneously [137,138].

Conventional 2D fluoroscopy (Figure 5) is the most popular method of acquiring data in trauma surgery, especially in cases of pelvic and acetabular trauma. The main benefit of the C-arm is its accessibility, being present in every hospital. Furthermore, the C-arm generates less radiation when compared with CT scans and 3D fluoroscopy. However, 2D fluoroscopy suffers from poorer image quality (especially the poorer delineation of anatomical structures) [139,140,141].

3D fluoroscopy represents a relatively new technology; it allows for 3D reconstruction by using multiple intraoperative fluoroscopy images. Unfortunately, this method requires an increased dose of radiation and patient exposure [142,143,144]. Compared with 2D fluoroscopy, 3D fluoroscopy gives better image quality. It is an increasingly popular alternative e.g., in acetabular and calcaneal surgery [142,145,146,147].

### 2.9. Imageless Navigation

This type of navigation is characterized by the fact that it does not require preoperative and postoperative images for planning and guiding surgery [148]. Instead of these procedures, joint kinetic information and bone morphology information are used for planning and to devise guiding maps. Regarding its applications in orthopedics, this kind of system was originally developed for TKA and THA applications [149,150].

**Figure 6:**
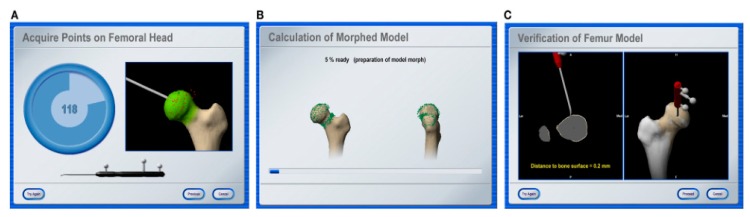
Process of the bone morphing: (**a**) landmarks acquisition, (**b**) calculation of the morphed model and (**c**) verification procedure of the final results [3].

DRFs are fixed to the pelvis, femur and tibia to find the locations and coordinates of the mechanical axes of the femur and tibia. In the subsequent step, the centers of the hip, knee and ankle are calculated. This calculation is performed for the centers of the relative motion of DRFs during the passive movement of each joint. A significant benefit of this type of navigation is that it does not require image registration. Femoral rotational alignment is adjusted by referring to the epicondylar line, the posterior condylar line or the sulcus line, which are determined by touching landmarks [151,152]. Tibial rotational alignment is adjusted by referring to the posterior tibial line or the tibial tubercle. This method is based on image morphing (Figure 6), which improves the accuracy and visual information of the image of the knee area [153]. This offers the best compromise taking into account ligament balancing and axial alignment. Regarding preoperative imaging and planning in THA, imageless navigation is a less costly and less time-demanding method when compared with CT navigation [154,155,156,157].

### 2.10. Ultrasound-Based Navigation

The application of ultrasound (US) systems for intraoperative guidance represents one of the most significant recent developments in CAOS systems. The main benefit of this type of system is its elimination of ionizing radiation exposure for both the surgical team and the patient [158]. Moreover, this system does not require tracking devices to be fitted to the patient. The next major issue is the modelling of small and tiny bones. By using ultrasound guidance, it is possible to perform 3D volume rendering of small bones and their fractures. In this way, it is possible to carry out accurate mapping of preoperative images and to devise a surgical plan for the purpose of real-time surgical guidance. Such a procedure can generally only be performed for large bones, including the femoral bone and pelvis [158,159].

In conventional CAOS systems, the preoperative model and surgical planning are registered between each other with respect to the patient in order to estimate the 3D transformation model between the preoperative and intraoperative images. From this perspective, the registration procedure is a crucial aspect of the entire system. Ultrasound computer-assisted orthopedic surgery (UCAOS) systems deal with the registration of intraoperative ultrasound images by means of the preoperative model [158,160].

**Figure 7:**
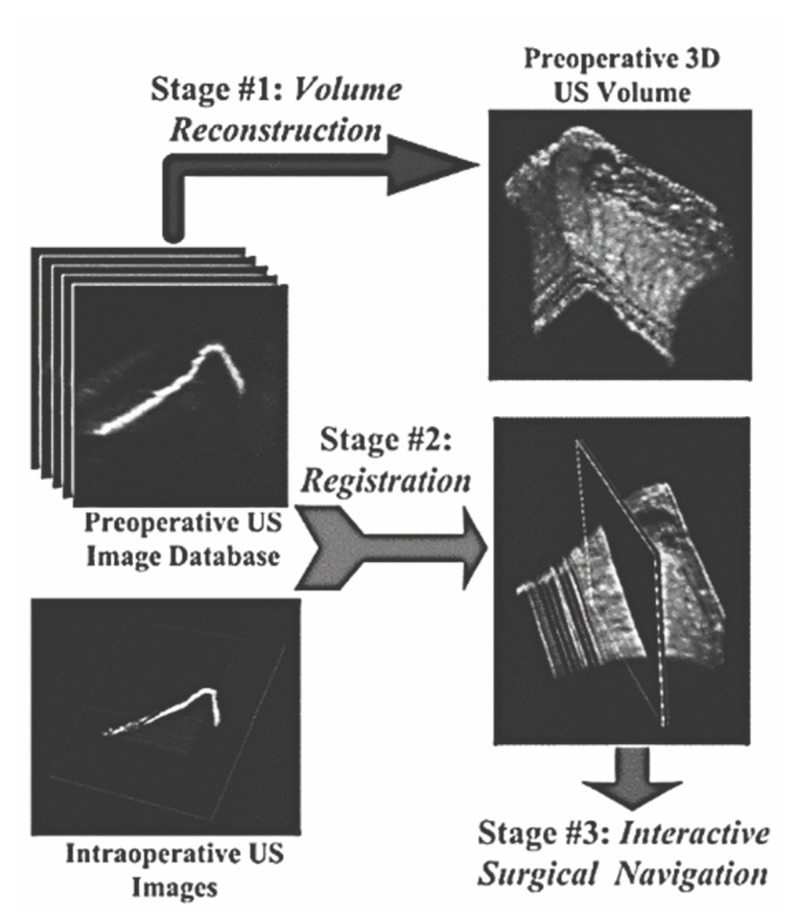
Structure of UCAOS system [158].

UCAOS systems are relatively novel and challenging alternatives for image-guided navigation. This hybrid type of system utilizes both preoperative and intraoperative images (Figure 7). Instead of CT images, ultrasound imaging (US) is used for preoperative images, and intraoperative fluoroscopy images are used for surgical guidance. In some cases, it is complicated to mount an invasive tracking device. For such cases, US preoperative images provide a reliable alternative for tracking the target position and coordinates of anatomical structures. An ultrasound probe is equipped with an optical target. This system is tracked by using a real-time video system in order to obtain real-time position information [160,161].

During the preoperative phase, a sequence of 2D US images are quired to obtain detailed information about the target anatomy, including its positional information. Based on this information, a preoperative database is created, generating a 3D volumetric representation of the target anatomy. During the intraoperative phase, the preoperative US volumetric images are registered with the intraoperative 2D US images [158].

UCAOS systems involve complex sets of equipment and methods, including an ultrasound probe, a calibration system, an ultrasound image acquisition system, an anatomical position tracker, a system for volume reconstruction, and visualization and registration algorithms [162].

### 2.11. Overall Comparison of Surgical Navigation

In this section, we compare pros and cons of individual reviewed surgical navigation techniques to highlight the limitations of individual types. The CT-based navigation is the most accurate. On the other hand, it takes a longer time for preoperative planning on CT images. This unfavorable fact increases the radiation exposure and costs. This phenomenon may be one of the limitations for surgeon acceptance. The imageless navigation does not require the CT images for localization. Instead of that, a DRF is directly attached to the pelvis, and consequently the pelvis coordinates are generated intraoperatively by using the anatomic landmarks. Some studies proved better accuracy of this technique for the cup alignment than conventionally used mechanical instruments [163,164,165,166]. In the case of the fluoroscopic navigation, it is used the same technique for placing the anatomic landmarks as in the imageless navigation. Nevertheless, the registration process is not suitable, and does not bring any advantages, when comparing with the imageless navigation [167]. The next limitation of the imageless navigation is accuracy for resurfacing by femoral deformity [168,169]. Ultrasound-based navigation brings a relevant alternative for the cases where it is complicated to mount the tracking device. This method is beneficial especially in the case of the small bones tracking. On the other hand, ultrasound lack of identification of some tissues. This fact is caused by a weaker contrast in the comparison with the CT and MRI imaging. We need to consider the fact that the ultrasound causes the thermal effect in the tissues passing through, also the ultrasound causes the mechanical effect, which is manifested by the mechanical pressure. These phenomena represent certain limitations of the ultrasound-based navigation. Ultrasound is a perfect tool for investigating the soft tissues, but not also reliable for the bones. Here, we are limited with a weaker contrast, and it reduces modeling preciseness of the bones [170].

### 2.12. A Role of Medical Robots in Orthopedic Assist Surgery

A challenging and perspective area in the orthopedic assist surgery is the use of medical robots. The concept of the medical robots for orthopedic surgery has been well known over 20 years. Robots allow for performing operations with better precision and repeatability, compared with conventional procedures. By definition, the medical robots are supposed to play an active role in the orthopedic surgery. They are described by two essential features. They can be used directly in the case of the bone cutting, or indirectly for placement or holding cutting jigs. The commonly knowns systems include automatic, semi-automatic and passive robotic systems. This classification is done on the base of the usage in the orthopedic surgeries. The passive systems need a direct manipulation by the surgeon. On the other hand, the automatic robotic systems perform the surgical tasks autonomously, without using the surgeon input. The semi-automatic systems have a certain level of autonomy, on the other hand, they require a certain surgeon action, like is the definition of the resection parameters. Robots are applied for placing the orthopedic implants, or for guiding the surgeons for positioning instruments more precisely.

In the case of the total joint arthroplasty (TJA), robots are efficiently applied since tibia and femur can be fixed to a specific location. Next area is the spine surgery. In this area, we can mention the surgical guiding system, helping surgeons to provide a minimally invasive incision within the spine surgery. Also, this system inserts the orthopedics implants more precisely in the case of the presence of a multiple fracture. The robots are used for unicondylar knee, patellofemoral and total knee arthroplasty. A popular area of the robots is also the orthopedic trauma. Here, the medical robots are used for assistance in the closed fracture reduction, reconstruction, and providing the surgical treatment remotely. With using of the preoperative images, the fracture reduction is performed with a minimal invasiveness. We can mention other benefits in the fracture care, including a precise guiding of the femoral nails, insert and manipulation with the distal interlocking screws and bolts.

One of the main general benefits of the robotic systems is providing more precise surgical operations, when comparing with conventional techniques in the tasks like the long bone fracture reduction or spinal deformity correction. The next benefit of these methods is an application for young surgeons, not having deep experience with the orthopedic operations. The results are reproducible, this fact significantly eliminate the human factor and variations in the surgical outcomes. The medical robots also bring a significant elimination of the radiation exposure, and the time reduction of the surgery. Despite many benefits of the robotic systems in the orthopedics, there are arising issues which need to be mentioned. One of these is a safe using of the robots within the surgical operation. In this context, the medical staff, operating with the robots must be properly trained and the regularized process must be established. Lastly, some studies show that there is not a significant difference between the radiological outcomes, comparing the robot-assist surgeries with the conventional surgeries [171,172,173,174].

## 3. CAOS Systems from the Biomechanical Perspective

From the biomechanical perspective, CAOS systems focus mostly on achieving appropriate interaction between the internal components applied during the surgery and the human tissue [162,175,176,177]. For an orthopedic surgeon, these components include complex-shaped implants, different types of internal screws, and percutaneous screws used for pinning during the surgery [178,179]. All these biomechanical components are generally applied with the CAOS systems that are currently in wide use. These components have a potential to improve the overall accuracy of orthopedic surgery, but it also creates a room for further improvement in technical surgical components such as internal stabilizing devices [180,181,182,183,184]. On this basis, the following paragraphs outline the history and development of internal rods, screws and plates, along with a description of the current situation with regard to these biomechanical orthopedic devices. As has been mentioned before, CAOS opens up the possibility for further improvements in these devices, which can be achieved by various methods including:

- 3D modelling using parametric modelling programs,
- structural analysis using the finite element method,
- experimental testing of internal fixation devices,
- parametric optimization of orthopedic tools.

These methods for the improvement and optimization of individual types of internal stabilization tools are discussed further below [185,186,187,188,189,190]. From the biomechanical perspective, the internal implants applied during CAOS can be divided into several types, including pedicle screws, Intraosseous screws, Schanz screws, cannulated screws, and Complex-shaped implants and plates.

### 3.1. Percutaneous Screws

This is an internal fixation method which has the advantage of minimizing the difficulty of surgery as an established mini-invasive procedure. From the perspective of the process, the surgical technique using these percutaneous screws can be divided into several steps:

- proposal for the application of fixation devices using various imaging techniques,
- application of a guide wire,
- measurement of the desired screw length,
- hole drilling for the screw insertion,
- application of the percutaneous screw.

From the mechanical perspective, the process described above is a workflow that can be further improved and optimized. Individual areas for potential improvement are outlined below [191,192,193,194,195].

From the perspective of materials, these types of components can be optimized through the application of bioabsorbable metals [196,197].

From the technological perspective, the methodology for the screw application has been improved substantially by the application of new imaging techniques, surgical methods and also surgical preparations of tools for the screw application. These techniques also decrease the X-ray exposure and improve the overall quality of the surgical process.

As can be seen in several investigations [198,199], the individual designs of internal screws are compared using finite element analysis. These studies may apply structural analysis in several ways. These include the use of a supercomputer for large-scale repetition of finite element analysis; extraction of smaller fixator sections and application of the loading to these details; evaluation of displacement under load; comparison of the application of different screws under the same screw loading conditions; analysis of the sacral fracture model in connection with internal fixation screws; and analysis of the ranges of motion for individual screws applied in the bone [200,201,202,203].

Other investigations supporting and verifying analytical results perform experimental evaluation through static testing, as described for instance in [204,205,206,207,208]. In these studies, the fixator is usually assembled together with a standardized or specific testing machine, axial or torsion load is applied, and individual results in different directions are measured. These studies also include fracture tests to determine the stiffness of individual screws.

### 3.2. Pedicle Screws

These types of screw are designed to correct deformities and treat trauma mostly in the lumbar spine [209,210]. Research in this field focuses mostly on the numerical analysis of design, with different dimensional, shape and material verifications, as can be seen in [210,211]. Nevertheless, increasing efforts are being made to design a 3D model based on radiographic 2D and 3D imaging. Based on these studies, a model of a deformed spine can be created by applying 3D modelling techniques. This can further improve the overall process of fixator design and optimization, as can be seen in past studies [211,212,213,214].

Another important aspect of improvements to pedicle screws is the evaluation of individual fixators with screws of different densities applied to the bone, as can be seen for example in [215,216]. The results can be analyzed to derive a flexibility curve. The results of these studies show that the optimal number of screws can be determined for an individual patient. This optimal number is further dependent on bone density, rigidity, the expected quality and size of the correction, etc. From this perspective, the effect of screw density on individual characteristics can be evaluated [215,217,218].

Pedicle screw densities can also be compared from the material perspective. As mentioned in [219,220], these orthopedic tools are experimentally evaluated, and their resulting strength is evaluated and analyzed, as are other mechanical properties such as fatigue life.

### 3.3. Intraosseous Screws

This technique is mostly used as a successful method of orthodontic treatment. Nevertheless, the technique still exhibits significant deficiencies which offer room for potential future improvements [221,222,223].

Another broad area of application can be found in orthopedics, prosthetics and osteosynthesis, as described in [224,225]. Among the important aspects connected with the use of these screws are the induction of stress shielding, biocompatibility, and corrosion resistance under stress. A comparison can be found in [226].

From the general perspective, this technique of fixation has been investigated by a large number of studies [226,227,228,229,230], but the orthodontic application of intraosseous screws still offers potential for further biomechanical investigation and technical improvements.

### 3.4. Schanz Screws

Schanz screws (along with Kirschner rods) are orthopedic components applied during external fixation—a successful method of treating long bone fractures (such as tibia fractures). These components have been developed over several decades, with the focus on optimizing material composition, shape and stiffness [231,232,233].

These screws have many applications in orthopedics, and as such they have been frequently evaluated and analyzed, as in [234,235,236,237,238]. Some recent studies also explore the potential future use of composite biodegradable materials for internal screws applied to the bone structure, or investigate nanocomposites applied to orthopedic devices [239,240,241].

These types of screw have been widely investigated by conventional methods, including analytical and experimental evaluation; future improvements are closely connected with current developments in material sciences such as carbon fibre composite printing.

### 3.5. Cannulated Screws

Cannulated screws are used in orthopedic treatment as part of a system for the fixation of fractures in osteotomies of larger and smaller bones—mainly in the treatment of hip, knee, ankle or foot injuries. Cannulated screws can be applied by means of guiding wires [242,243,244]. Although this method is increasingly used nowadays, for some types of surgery it still remains controversial. Studies of cannulated screws published in recent years usually focus on the same areas as studies of other screw types (mentioned above). These areas include the identification of screw density and optimal position in relation to the fractured bone, finite element method analysis for the accurate determination of the depth of the hole drilled into the bone, the suitability of cannulated screw insertion depending on mechanical bone properties in elderly patients, suitable timing and force of cannulated screw pullout based on bone healing status, and the experimental and numerical verification of tests [245,246,247,248,249]. There therefore exists a broad range of external fixation methods which offer potential for further biomechanical investigation and improvements of current solutions from a mechanical perspective.

### 3.6. Complex-Shaped Implants and Plates

This type of internal fixation involves the latest internal fixation devices, which require advanced manufacturing, 3D design and numerical analysis techniques. In addition to these technologies and methods, this type of fixation also requires the appropriate application of results from imaging and bone state analysis. The combination of all this information can enable the development of successful treatment devices designed for the specific individual conditions of a given fracture [250,251,252,253,254].

### 3.7. Materials of Orthopedic Implants

Materials for the orthopedic implants play an important role in the fixation process. The selection of a suitable implant material can influence the implant rigidity, corrosion, tissue receptivity and also biocompatibility [255]. An ideal implant material for the orthopedic purposes may be outlined with the following characteristics:

- chemically inert,
- completely biocompatible,
- higher strength,
- high resistance against fatigue,
- complete corrosion-proof,
- inexpensive.

Currently, there are three main categories of the materials for the prosthetic devices, including metals, polymers and ceramic. Metals are used for the orthopedic implants include alloys of surgical grade stainless steel, cobalt-chromium (Co-Cr) and pure titanium (Ti) and also alloys based on the titanium. Another conventionally used material is polymer. The most frequently used polymer in the orthopedics is ultra-high molecular-weight polyethylene (UHMWP) or high-density polyethylene (HDP) [256,257]. One of the most significant limitations of the polymers is relatively slow, temperature-dependent deformation and also the progressive wear—in the case of polyethylene. The last category of the materials is ceramic. The ceramic used for the orthopedics implants include aluminum oxide and calcium phosphates. A typical property of these materials is a resistance against compression on the other hand they are weak under tension and shear, and brittle [255,258].

## 4. Discussion and Future Perspectives

The purpose of CAOS systems is to eliminate inaccuracies from radiological results and at the same time to improve the accuracy of surgical procedures. These approaches make minimally invasive surgery easier and enable new surgical possibilities to be explored. CAOS systems are also ideally suited for training purposes. Another important aspect of CAOS is preoperative computer planning. This procedure allows for simulations of the planned surgery; the simulation makes it possible to optimize and improve the surgical plan. Although the delivered dose of intraoperative radiation is reduced by using volumetric image-based navigation, some approaches require increased surgical invasiveness; an additional operation is needed for the implantation of the markers. This logically prolongs the surgery and leads to increased blood loss. From this perspective, it is beneficial to use fluoroscopic image-based navigation as it reduces both the radiation dose and the operating time. Among the main benefits of CAOS systems is the reduction of invasiveness through the use of new instruments and new methods for image processing—including image optimization to boost specific tissues while others may be suppressed, or the use of segmentation tools allowing for the extraction of the bones or other objects of interest. Objects of interest are often supplemented by video sequences. This procedure allows for some procedures to be performed less invasively by using a small manipulator.

One recent trend in this area focuses on using virtual reality technologies, which have the potential to improve the accuracy of navigation. These technologies are also applicable in surgical training and rehabilitation. Virtual surgical tools and components can significantly reduce the duration of surgical procedures such as the appropriate selection of modular parts for THA. Conventional methods in surgical training using the master-apprentice training are inefficient. We should note that the orthopedics is highly depended on the technical skills. Orthopedics training simulators based on the virtual reality have a great potential to improve the surgeon skills. By using the virtual reality-based simulators, we can emulate the surgical environment and do testing of the surgical procedures without serious impact on the patients. Training based on the virtual reality may be also beneficial for the medical residents. By the term residency, we refer to a stage of graduate medical training. Residents, who are trained with the virtual reality perform surgery in a comparative reduced time, against those with the conventional training.

Regarding the mechanical components of CAOS systems, internal stabilizing rods and screws have been investigated in considerable detail. This is largely due to the number of these products that are widely applied in orthopedics, as well as the fact that from the mechanical or biomechanical perspectives, these screws are less complicated structures for standard structural investigation. Mechanical and material scientists should therefore direct their future research towards the application of current developments in mechanics and materials and the evaluation of these improvements. Another potential avenue for future research lies in enhanced cooperation among surgeons, radiologists, imaging specialists, material scientists and biomechanical scientists; this can potentially lead to more detailed and more complex descriptions and improvements of internal fixation methods. In this context, the multidisciplinary cooperation in CAOS systems is essential. Image specialists are mostly aimed on developing systems based on the artificial intelligence for an autonomous recognition of the bones and other structures of interest. By using the methods of virtual reality and image processing, these systems are aimed to emulate the mechanical components. Optimization of geometrical features and mechanical design of these mechanical components are task for the mechanical specialist. Another issue of the mechanical components in the CAOS systems is selection and development of optimal materials. For instance, the current trend lead to development of non-magnetic and biocompatible materials which are not limited within examination in MRI. Other element of the CAOS systems deals with incorporating surgeons and other clinical specialists and their insights from the clinical point of the view. As surgeons have special needs for specific surgical operations, technical specialists need to optimize the CAOS solutions for specific applications [259,260,261,262].

The main benefit of the CAOS systems is the outlier’s elimination from the radiologic images by enhancing the preciseness of the surgical procedures. In the next step it is mini invasiveness of the surgery and mainly exploring new possibilities and concepts in the orthopedics surgeries. Lastly, it is utilization of the CAOS systems for education and training of new physicians. Preoperative planning enables perform simulation of surgery, and consequently brings optimization of the surgical planning. Despite that, it is possible to reduce the intraoperative radiation exposure by using volumetric-based navigation, and by using the robotic milling it is possible to significantly reduce the volume of intraoperative pulmonary embolism, some methods increase the surgical invasiveness. This is caused with the requirement of additional operation to place and implant the markers. This also causes a prolongation of the surgical operation duration, and in the same time it increases the blood loss. In this context, fluoroscopic navigation appears to be suitable due to the reduction the radiation dosage and surgical time. By integrating new methods, instruments, image hybridization and visualization possibilities, the CAOS technologies have a strong future potential to reduce invasiveness. A significant breakthrough is the CAOS systems is supposed to be integration of the technologies based on the augmented reality, including the overlay displays and binoculars and systems for laser beam guidance. An important role of the CAOS system is supposed to be in new hip and knee implants which are supposed to have a smaller size when comparing with the regular ones. Another issue is cartilage replacement and simultaneous ligament reconstruction within one single surgical operation by requiring only one tiny incision. The CAOS systems also have an ambition to be used as a system with a minimal invasiveness in the tissue engineering on the artificial cartilage replacement. In this operation, the CAOS systems can control the tissue shape [263,264,265,266,267,268].
